# Supplementary material for: The Evolution and Characterization of the RNA Interference Pathways in Lophotrochozoa
Source: Genome Biol Evol. 2024 May 7;16(5):evae098. doi: 10.1093/gbe/evae098 (PMC11114477; doi:10.1093/gbe/evae098)
Supplement: evae098_Supplementary_Data [file evae098_supplementary_data.zip › Supplementary_materials.zip!/selected_miRNAs/NoGe_selected_miRNAs/novel_miRNA_families_pdf_files/NMRB01000201.1_Notospermus_geniculatus_strain_Ushimado_scaffold201,_whole_genome_shotgun_sequence_7102.pdf]

| | | |
|---|---|---|
| Provisional ID | : | NMRB01000201.1_Notospermus_geniculatus_strain_Ushimado_scaffold201,_whole_genome_shotgun_sequence_7102 |
| Score total | : | 1320.2 |
| Score for star read(s) | : | 3.9 |
| Score for read counts | : | 1312.4 |
| Score for mfe | : | 0.8 |
| Score for randfold | : | 0 |
| Score for cons. seed | : | 3 |
| Total read count | : | 2586 |
| Mature read count | : | 2492 |
| Loop read count | : | 0 |
| Star read count | : | 94 |

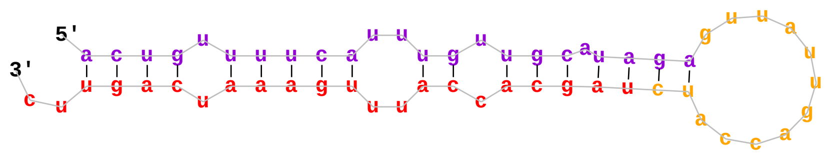

Genome coordinates: NMRB01000201.1 523621-523679

pre-miRNA sequence:
ACTGTTTTCATTTGTTGCATAGAACTGTTTTCATTTGTTGCATAGAGTTATTGACCATCTAGCACCATTTGAAATCAGTTC

Mature sequence: TAGCACCATTTGAAATCAGTTC

BLAST results for the pre-miRNA sequence against the *Lineus longissimus* genome assembly:

| **Scaffold** | **Start** | **Stop** | **Identity** | **e-value** |
|---|---|---|---|---|
| OU343002.1 1 | 3549584 | 3549568 | 100.000 | 2.2 |

```
Nge_novel_miRNA          ACTGTTTTCATTTGTTG
Llo_genome               ACTGTTTTCATTTGTTG
```
